# The clinical significance of circulating miR-21, miR-142, miR-143, and miR-146a in patients with prostate cancer

**DOI:** 10.5937/jomb0-32046

**Published:** 2022-04-08

**Authors:** Ibrahim Murat Bolayırlı, Bülent Önal, Mutlu Adıgüzel, Dildar Konukoğlu, Çetin Demirdağ, Eda Merve Kurtuluş, Fethi Ahmet Türegün, Hafize Uzun

**Affiliations:** 1 Istanbul University-Cerrahpasa, Cerrahpasa Faculty of Medicine, Department of Medical Biochemistry, Istanbul, Turkey; 2 Istanbul University-Cerrahpasa, Cerrahpasa Faculty of Medicine, Department of Urology, Istanbul, Turkey; 3 İstanbul Gelişim University, Faculty of Health Sciences, Department of Nutrition and Dietetics, Istanbul, Turkey; 4 Istanbul Atlas University, Faculty of Medicine, Department of Medical Biochemistry, Istanbul, Turkey

**Keywords:** Prostate cancer, miR-21, miR-142, miR-143, miR-146a, rak prostate, miR-21, miR-142, miR-143, miR-146a

## Abstract

**Background:**

Prostate cancer (PCa) is the most common type of solid tissue cancer among men in western countries. In this study, we determined the levels of circulating miR-21, miR-142, miR-143, miR-146a, and RNU 44 levels as controls for early diagnosis of PCa.

**Methods:**

The circulating miRNA levels in peripheral blood samples from 43 localized PCa patients, 12 metastatic PCa (MET) patients, and a control group of, 42 benign prostate hyperplasia (BPH) patients with a total of 97 volunteers were determined the by PCR method.

**Results:**

No differences in the DCT values were found among the groups. In PCa and PCaMet groups the expression of miR21 and miR142 were higher compared to the BHP group. No other differences were observed among the other groups. miR21 expression in the PCa group was 6.29 folds upregulated whereas in the PCaMet group 10.84 folds up-regulated. When the total expression of miR142 is evaluated, it showed a positive correlation with mir21 and mir 146 (both p<0.001). Also, the expression of miR146 shows a positive correlation with both miR21 and miR143 (both p<0.001). Expression of miRNAs was found to be an independent diagnostic factor in patients with Gleason score, PSA, and free PSA levels.

**Conclusions:**

Our study showed that co-expression of miR21, miR-142, miR-143, and miR-146a and the upregulation of miR-21 resulted in increased prostate carcinoma cell growth. In the PCaMet group, miR21 is the most upregulated of all miRNAs. These markers may provide a novel diagnostic tool to help diagnose PCa with aggressive behavior.

## Abstract (sr)

**Uvod:**

Karcinom prostate (PCa) je najčešći tip raka čvrstog tkiva među muškarcima u zapadnim zemljama. U ovoj studiji, odredili smo nivoe cirkulišućih miR-21, miR-142, miR-143, miR-146a i nivoe RNU 44 kao kontrole za ranu dijagnozu PCa.

**Metode:**

Nivoi cirkulišuće miRNA u uzorcima periferne krvi određivani su PCR metodom kod 43 pacijenta sa otkrivenim PCa, 12 pacijenata sa metastatskim PCa (MET) i kao kontrolnom grupom kod 42 pacijenta sa benignom hiperpla zijom prostate (BPH) u ukupno 97 dobrovoljaca.

**Rezultati:**

Nisu pronađene razlike u vrednostima DCT među grupama. U grupama PCa i PCaMet ekspresija miR21 i miR142 bila je veća u poređenju sa BHP grupom. Nisu uočene druge razlike među ostalim grupama. Ekspresija miR-21 u grupi PCa bila je 6,29 puta lošije regulisana, dok je u grupama PCaMet regulacija bila povećana 10,84 puta. Kada se proceni ukupna ekspresija miR-142, ona je pokazala pozitivnu korelaciju sa miR-21 i miR-146 (oba p<0,001). Takođe ekspresija miR-146 pokazuje pozitivnu korelaciju sa oba miR-21 i miR-143 (oba p<0,001). Utvrđeno je da je ekspresija miRNA nezavisan dijagnostički faktor kod pacijenata da Gleason skorom, i nivoima PSA i slobodne PSA.

**Zaključak:**

Naša studija je pokazala da je ko-ekspresija miR-21, miR-142, miR-143 i miR-146a i povećana regulacija miR-21 rezultirala povećanjem rasta ćelija karcinoma prostate. U grupi PCaMet, miR-21 je bio najbolje regulisan od svih miRNA. Ovi markeri mogu pružiti novi dijagnostički alat koji će pomoći u dijagnostici PCa sa agresivnim ponašanjem.

## Introduction

In western countries, prostate cancer (PCa) is the most common solid tumor in men [1]. In 2018, the number of newly diagnosed patients with PCa reached 1.3 million [2]. As the incidence of PCa increases in aging males, especially the eighth decade of life shows malignant changes in > 70% of individuals according to autopsy reports. The annual mortality load of PCa is 220,000 deaths, making it the sixth leading cause of cancer mortality among men [3]. PCa mostly arises from the peripheral zone of the gland. Epidemiological studies showed that having a first-degree relative with PCa increased risk for an individual by approximately two-to three-fold [4]. The incidence of PCa in African-American men is higher when compared to White men [5].

Prostate biopsy is the gold standard for the diagnosis of PCa. Samples are taken bilaterally from apex to base, as far posterior and lateral as possible in the peripheral gland. While for small-sized prostates at least 8 systematic (~30cc) biopsies are required; for larger prostates, 10 to 12 biopsy samples are required which eventually increases the health care cost. Prostate-specific antigen (PSA), a serine protease inhibitor is produced by both malignant and non-malignant epithelial cells. Since the 1990s, it has been used as a screening test. The use of PSA as a screening test has led to the increased detection of the early stage of cancer and a fall in the incidence of metastatic disease, also a reduction in related mortality. Over detection of PCa leads to overtreatment, increased side effects, complications, patient anxiety, and high costs [6]. Although PSA is prostate-specific, it increases not only in PCa but also and prostatitis. Due to these characteristics, the use of new biomarkers with high sensitivity and specificity ratios was considered for the early detection of PCa.

MicroRNAs (miRNAs) are a single-chain, endogenous, highly conserved group of small, noncoding RNA groups of about 19–25 nucleotides in length [7]. miRNAs have been shown to have crucial roles in certain biological processes and pathological conditions. miRNAs appear as important cytoplasmic regulators of gene expression. miRNAs act as post-transcriptional regulators of their messenger RNA (mRNA) targets via mRNA degradation and/or translational repression [8]. In recent years, miRNAs were used for defining the physiopathology of cancer and various diseases. The human genome contains more than 1000 miRNAs, and estimates indicate that some 60% of the human protein-coding genes may be regulated by miRNAs, which means they may sig nificantly affect the expression of several proteins [9].

On the other hand, miRNAs play a role in many cellular and biological processes such as cellular differentiation, proliferation, apoptosis, erythropoiesis, fibrosis, angiogenesis, and immunity. In some cancers, some miRNAs function as oncogenes others functions as tumor suppressor genes, indicating that miRNAs regulate tumor progression, metastasis, and invasion [10]. In addition to their potential as tissuebased markers for cancer classification, circulating miRNAs in the blood of cancer patients might be used as potential diagnostic and prognostic biomarkers [11]. A recent study showed that miR-21 promotes hormone-dependent and hormone-independent growth in PCa [12]. MiR-143 has been shown to have a strong relationship with PCa [13]. It was shown that the expression of miR-146 was induced in mice with PCa [14].

Our study aimed to investigate the pattern of cancer-associated miRNAs, miR-21, -142, 143, and -146, and as a control RNU43 miRNA in the plasma of PCa patients with local/metastatic disease and patients with benign prostate hyperplasia (BPH).

## Materials And Methods

### Subjects

Ninety-seven men, who were admitted to the Outpatient Clinic of Urology Department, Cerrahpasa Medical Faculty of Istanbul University-Cerrahpasa were included in our study. The patients were divided into three groups as 43 patients with localized PCa, 12 patients with metastatic PCa (MET), 42 patients with BPH as a control group. All subjects gave their informed consent before participating in the study. The study design was approved by the Ethical Committee of Cerrahpasa Medical Faculty (14/07/2016-256677) and was conducted in conformity with the Declaration of Helsinki. The complete medical history, physical examination, laboratory in vestigation, and clinicopathological features were obtained and recorded for all patients. Tumor staging was performed in conformity with the American Joint Committee on Cancer (AJCC) system, 7th edition tumor, lymph nodes, metastasis (TNM) staging classification.

According to the result of prostate biopsy, patients diagnosed with PCa and BPH were enrolled in our study. Patients who had undergone surgery, chemo/radiotherapy, and patients with secondary malignancy, acute infections, diabetes mellitus, hypertension, kidney diseases, and rheumatologic diseases were excluded from the study.

All patients underwent at least a 12-core biopsy at our institution due to increased prostate-specific antigen serum levels (>4 ng/mL) and/or suspicious findings on digital rectal examination. Samples are taken bilaterally from apex to base, as far posterior and lateral as possible in the peripheral gland. The tumors were graded according to the modified Gleason grading system [15] and staged according to the guidelines [16].

### Collection of blood samples

Venous blood samples were collected into plain tubes and tubes which were coated inside with ethylenediaminetetraacetic acid (EDTA), in the morning after overnight fasting (10–12 h). The plain tubes were centrifuged for 10 minutes at 4000 rpm at 4°C.

### Biochemical analysis

Extraction of small RNA molecules from human blood serum was performed by the EXTRACTME miRNA KIT (BLIRT, Poland) according to the manufacturer's instructions. The RNA samples were immediately stored at -80°C until they were reverse transcribed into cDNA. Firstly, the miRNA samples were transcribed into complementary DNA (cDNA) using a cDNA Synthesis Kit (High Capacity) (Wizbio Solutions, Korea). The reaction was carried out at 25°C for 10 min, 37°C for 120 min, and 85°C for 5 min on a SimpliAmp Thermal Cycler (Thermofisher, USA). The cDNA samples were stored at -20°C until used.

Quantitative Real-Time PCR kits made specifically for accurate miRNA analysis were used to evaluate the expression of the following miRNAs from serum samples: miR-21, miR-142, miR-143, miR-146a, and RNU44 (endogenous control). For Real-Time PCR, Amplifyme Sg Universal Mix (Blirt, Poland) was used. The primers used in the study were obtained from Suarge Biyoteknoloji (Istanbul, Turkey) (Table 1). Each sample was tested in triplicate on a real-time PCR system (Step One Plus real-time PCR system, Applied Biosystems, Carlsbad, CA, USA). The RT-PCR reaction was performed at 95°C for 10 min, followed by 40 cycles at 95°C for 15 seconds and 60°C for 1 min. The expression levels of miR-21, miR-142, miR-143, miR-146a were normalized to RNU44 were calculated using the 2-ΔΔCt method [17].

**Table 1:** Reverse transcription oligos specific to miRNAs

Primers	Sequence
miR-142 RT	GAAAGAAGGCGAGGAGCAGATC- GAGGAAGAAGACGGAAGAATGT- GCGTCTCGCCTTCTTTCTCCATAAA
miR-143 RT	GAAAGAAGGCGAGGAGCAGATC- GAGGAAGAAGACGGAAGAATGT- GCGTCTCGCCTTCTTTCACCAGA- GA
miR-146a RT	GAAAGAAGGCGAGGAGCAGATC- GAGGAAGAAGACGGAAGAATGT- GCGTCTCGCCTTCTTTCAACCCATG
miR-21 RT	GAAAGAAGGCGAGGAGCAGATC- GAGGAAGAAGACGGAAGAATGT- GCGTCTCGCCTTCTTTCTCAACATC
RNU44 RT	GAAAGAAGGCGAGGAGCAGATC- GAGGAAGAAGACGGAAGAATGT- GCGTCTCGCCTTCTTTCAGTCAGTT
miR-142 Forward	GCGGTGTAGTGTTTCCTACT
miR-143 Forward	GGTGCAGTGCTGCATCT
miR-146a Forward	GGCCTGAGAACAGAATTCCAT
miR-21 Forward	GCGGTAGCTTATCAGACTGATGT
RNU44 Forward	CCTGGATGATGATAAGCAAATG
Universal Reverse	CGAGGAAGAAGACGGAAGAAT

Serum PSA levels were determined by electrochemiluminescence immunoassay on the Roche Modular Analytics E 601 immunoassay analyzers. For PSA, inter-and intra-assay coefficients of variation (CV) values were <10%.

### Statistical Analysis

For statistical analysis, SPSS 21.0 software package (SPSS Inc., Chicago, IL, USA) was used. Descriptive statistics such as age, free PSA, PSA levels were given in mean and median. For expression test of normality was followed by the Kruskal Wallis test for comparison. For post hoc comparison within significantly different groups, Mann Whitney U test was performed. Stepwise regression analysis was applied to determine the independent effects of expressions and to reveal the relation Spearman's test was used. The statistical significance level was p<0.05.

## Results

Age, free PSA, and total PSA levels of the groups are given in Table 2. No differences in agePSA levels in the PCaMet group were also higher than BPH (p<0.001) and PCA groups (p<0.01). According to the Gleason score, PCaMet also showed a higher mean compared to the PCa group (p<0.05).

**Table 2:** Demographic characteristics and pathological findings of the BPH, PCa, PCaMet groups BPH, benign prostate hyperplasia; PCa, prostate cancer; MET, metastatic PCa.<br>Comparison with BPH a= p<0,001 ; a1=p<0.01<br>Comparison with PCa,b=p<0,001; b1=p<0,05 b2=p<0,01<br>*Kruskal-Wallis; **Mann Whitney U

	Age (years)	FreePSA (ng/mL)*	PSA*	Gleason’s Score**
BPH (n: 42)	64.33±7.04	0.89±0.60	4.99±2.66	
PCa (n: 43)	65.55±6.69	1.52±0.91^a ^	8.68±6.21^a^	6.37±0.61
PcaMet (n: 11)	66.45±4.69	1.97±0.53^a,b1^	16.04±8.48^a,b2 ^	7.00±0.10^b ^

PSA levels in the PCaMet group were also higher than BPH (p<0.001) and PCA groups (p<0.01).According to the Gleason score, PCaMet also showed a higher mean compared to the PCa group (p<0.05).

When the average CT value of RNU44, which is the reference miRNA, is subtracted from the CT values of the groups, the ΔCT values are obtained and these values are given in Table 3. No differences in the ΔCT values (Table 2) were found among the groups (p> 0.05)

**Table 3:** Analysis of DCT values of Benign Prostate Hyperplasia; Prostate Cancer; and Metastatic PCa groups BPH, benign prostate hyperplasia; PCa, prostate cancer; MET, metastatic PCa.<br>*Kruskal Wallis

	BPH*		PCA*		MET*	
miR-21	32.18	±2.78	32.54	±2.54	31.65	±3.05
miR-142	32.83	±3.31	30.86	±5.74	32.15	±3.06
miR-143	24.45	±12.92	25.58	±12.34	28.21	±11.91
miR-146	31.37	±9.37	32.36	±8.07	34.40	±2.07

In Table 4, the mean of ΔΔCT values is given. In PCa and PCaMet groups the expression of miR-21 and miR-142 were higher compared to the BHP group. No other differences were observed among the other groups. In Table 5 data are presented as fold changes derived in terms of the mean 2^−ΔΔCT^ method. According to Table 5; miR-21 expression in the PCa group was 6.29 folds upregulated whereas in the PCaMet group 10.84 folds up regulated. miR-142 expression was 10.98-fold up-regulated in the PCa group and 9.24-fold upregulated in the PCaMet group. In Table 6, correlations of the miRNAs were given. When the total expression of miR-142 is evaluated, it showed a positive correlation with miR-21 and mir 146 (both p<0.001). Also, expression of miR-146 shows a positive correlation with both miR-21 and miR-143 (both p<0.001). The stepwise analysis results show that none of these markers are good at distinguishingly predicting the PSA level. And age, PSA, free PSA levels are independent of miR-21, miR-142, and miR-143 expression.

**Table 4:** Comparison of ΔΔCT Values of Benign Prostate Hyperplasia; Prostate Cancer; and Metastatic PCa groups. BPH, benign prostate hyperplasia; PCa, prostate cancer; MET, metastatic PCa.<br>Comparison with BPH a= p<0.005<br>Comparison with PCa b=p<0.005

	BPH	PCa	PCaMet
miR-21	-2.42±0.37	0.95±3.95^a ^	0.02±4.41^a^
miR-142	-1.96±0.13	-0.02±3.30^a^	0.07±3.80^a ^
miR-143	1.05±0.05	0.70±2.75	2.27±1.98
miR-146	1.46±0.23	-0.19±1.97	-0.35±2.79

**Table 5:** Comparison of the fold changes of miRNA-143, 146, 142 and miR-21 PCa, prostate cancer; MET, metastatic PCa.<br>Comparison with BPH group a=p<0.005<br>Comparison with RNU44 b=p<0.005

Group	miRNA143	miRNA146	miRNA142	miR21
PCa<br> (n=45)	2.92^a^	3.8489^a^	10.0838^a^	6.294^b^
PcaMet<br> (11)	0.37^a^	5.5250^a^	9.2418^a^	10.8464^b^

**Table 6:** Correlation table for the miRNA expressions PSA, Prostate specific antigen<br>**p<0.001 *p<0.05

	FreePSA	PSA	ΔCtmiR142	ΔCtmiR21	ΔCtmiR146	ΔCtmiR143
FreePSA		0.843**	-0.134	0.022	-0.172	-0.142
PSA	0.843**		-0.088	0.021	-0.156	-0.077
ΔCt mir142	-0.134	-0.088		0.751**	0.685**	0.530
ΔCt mir21	0.022	0.021	0.751**		0.780**	0.103
ΔCtmir146	-0.172	-0.156	0.685**	0.780**		0.645**
ΔCtmir143	-0.142	-0.077	0.530	0.103	0.645**	

## Discussion

In recent studies, miRNAs have been used as a predictive tool in many cancer types such as prostate, breast, ovarian, colon, pancreas, and lung. The serum expression level of miRNAs varies according to the cancer type, the diagnosis of the disease can be made early and treatment can be started and important information can be obtained about the prognosis [18] [19] [20] [21] [22] [23] [24] [25] [26] [27] [28]. In the current study, we have found a significant difference between BPH and PCa in the expression of miR-21. In addition, when PSA levels were divided into groups, the PSA value of the groups (approximately 76%) had 2.5–9.9 ng/mL miR-21 and miR-143 expressions were significantly different among the groups. A negative correlation was found between miR-21 with oncogenic function and free PSA only in the PCa group. However, the expression of this miRNA was found to be an independent diagnostic factor in patients with Gleason scores. These results showed that changes (especially increases) in miR-21 expression can be effective in the transition to invasive potential. miR-21 can contribute to prostate cell transformation in PCa.

Although the current gold standard of PCa diagnosis is the prostate biopsy, this sampling technique is susceptible to misdiagnosis and a negative biopsy cannot fully rule out cancer [29]. Circulating PSA is currently the most common non-invasive biomarker used to detect PCa, despite the controversies around its use as a screening tool [18]. On the way to better tools, miRNAs can be a non-invasive marker. Zhang et al. [28] reported that serum miR-21 expression is upregulated in patients with hormone-refractory PCa, which is resistant to docetaxel-based chemotherapy. Serum miR-21 levels have been also shown to correlate with serum PSA. Upregulation of miR-21 is a critical event in the development of metastasis and invasion of PCa in immunocompromised mice [30]. Bertoli et al. [31] reported a group of miR-21 is commonly deregulated in extracellular fluids of PCa patients. Kurul et al. [20] observed that miR-21 was overexpressed in patients with low-risk PCa. Unlike other studies, Folini et al. [32] found no difference in terms of miR-21 expression between PCa and normal tissue. Liu et al. [33] demonstrate that miR-21 induces tumor angiogenesis through targeting PTEN, leading to activate AKT (also known as Protein Kinase B) and extracellular signal-regulating kinase 1/2(ERK1/2) signaling pathways, and thereby enhancing hypoxia-inducible factor-1α (HIF-1α) and vascular endothelial growth factor (VEGF) expression; HIF-1α is a key downstream target of miR-21 in regulating tumor angiogenesis in human PCa cells. Li et al. [34] suggested that miR-21 could promote apoptosis resistance, motility, and invasion in prostate cancer cells and these effects of miR-21 may be partly due to its regulation of PDCD4, TPM1, and MARCKS. Genetherapy using miR-21 inhibition strategy may therefore be useful as a PCa therapy. In our study,overexpression of miRNA-21 was approximately observed in half of the patients. miR-21 expression of group C (PSA=10–19.9 ng/mL) is significantly higher than group A (PSA <2.5 ng/mL).

MiR-21 expression of group D (PSA>20 ng/mL) is significantly higher than group C (PSA=10–19.9 ng/mL). High levels of expression of miR-21, which was found to function as an oncogene, have been observed in hematological malignancies such as AML, CLL, and glioblastoma, and in many cancer types of solid tumors such as prostate and thus, miR-21 is transcriptionally activated by Stat3 in the IL-6 signaling pathway [35]. miR-21 has been well characterized in invasion and metastasis events. miR-21 promotes cell movement and invasion by targeting the mRNA of PTEN, a tumor suppressor protein [32] [36] miR-21 expression in PCa tissue samples was significantly associated with pathological stage, lymph node metastasis, capsularinvasion, organ-confined disease, Gleason score, biochemical recurrence, and patient follow-up. The miR-21 expression could also be an independent predictor of biochemical recurrence [25]. In our study, miR-21 was shown to be the most common of the over-expressed oncomiRs in PCa as in other cancers. In the PCaMet group, miR-21 is also the most upregulated of all miRNAs. Expression of miR-21 showed a positive correlation with both miR-146 and miR-143. Accordingly, gene therapy using miR-21 inhibition strategies may prove useful for PCa therapy [25]. miR-21 is also helpful as a biomarker to predict cancer progression and miR-21 promotes tumor invasiveness and induces castration-resistance phenotype in PCa [37].

MiR-142-3p plays multiple roles in human cancers. miR-142-3p was upregulated in PCa tissues and cell lines relative to non-tumor samples and normal prostate cells. miR-142-3p levels were negatively correlated with forkhead box transcription factor O1 (FOXO1) in PCa and confirmed that miR-142-3p repressed FOXO1 expression through binding to the 3 UTR of FOXO1 mRNA [38]. Barceló et al. [39] by using miRNA-based models states that evaluation of miRNA expression and PSA levels together, might increase the classification function of the PSA screening test with diagnostic and/or prognostic potential: (PSA + miR-142-3p + miR-142-5p + miR-223-3p) model to discriminate PCa from BPH; and (PSA + miR-342-3p + miR-374b-5p) model to discriminate between GS 7 tumors and men presenting PSA 4 ng/mL with no cancer or GS6 tumors.

The pathway analysis of predicted miRNA target genes supports a role for these miRNAs in the etiology and/or progression of PCa diagnostic biomarkers in semen exosomes. Baffa et al. [40] show that when the results obtained from primary and metastatic bladder tumors were compared, it was determined that miR-143 expression decreased, however, miR-142-5p expression level increased.

In another study conducted with serum samples of 25 metastatic PCa patients, an increase in expression levels of miR-143 was found [41]. Another study by Barceló et al. [42] obtained that a clinically useful semen plasma miRNA-based combined model (PSA+miR-142-3p+miR-223-3p+miR-93-5p), which improves PCa specificity of the PSA test, for, firstly, predicting the presence of malignant tumors in a sample from the total population and secondly, and more interestingly for clinicians, for predicting PCa in samples from the positive PSA screening test (PSA>4 ng/mL). miR-143 expression in PCa tissue is significantly higher compared with expression in adjacent non-cancerous tissue, indicating an association between the molecule and the development of the disease [43] [44]. Transfection of miR-143 induces the apoptosis of PCa LNCap cells by down-regulating Bcl-2 expression [45]. In our study, miR-142 expression was suppressed by 53.3% in the BPH group and 33.3% in the PCa group. A positive correlation between miR-142 with miR-21 and miR-146 was found in all groups. When stepwise regression analysis was performed, the expression levels were affected by miR-142, and miR-146a was found to be independent of miR-21. Regression analysis showed that when the dependent variable miR-21 was taken, mir-142 and miR-146 were dependent on expression levels. There are also some regulatory roles attributed to miR-146a in PCa [46] [47].

Lin et al. [48] determined that the conditions with Gensini score> 7 were accompanied by the decreasing expression of miR-146a. When androgen-independent PCa cell lines (LNCaP-C81, LNCaP C4-2B, and PC3) were compared with androgen-sensitive cell lines (LNCaP and PC3-AR9), a decrease in miR-146 gene expression was observed in androgen-independent PCa cell lines. Moreover, the miR-146 loss has also been found to cause an aggressive course of PCa with an increase in multiple prometastatic proteins (ROCK1 and CXCR4) [48]. Hsa-miR-146a expression was decreased in Ta tumors of urothelial carcinomas [49]. It has been shown that miR-146a down-regulated may be due to increased EGFR signaling and may lead to aggressive PCa progression [50]. In the study of Mihelich et al. [51], 50 men with 100% Gleason grade 3 (low grade) PCa, 50 men with 30–90% Gleason grade 4 and/or 5 (high grade) PCa, and 50 patients with BPH, 16 miRNAs were detected in serum. miR-146a, one of which was detected at low levels in the high-grade PCa group. However, higher, and more heterogeneous levels were significantly detected in patients with low-grade PCa or BPH. It seems that our study is compatible with the existing studies in the literature. In PCa, miR-146a acts as a tumor suppressor gene.

As with many diseases, accurate and timely diagnosis of the disease is as vital as the application of appropriate and effective treatment in PCa. Although overexpression of miR-21 does not allow it to be used in the differential diagnosis of BPH, metastasis, and PCa, anti-miR-21 developed against miR-21 overexpressed in tissues and cells may be an effective treatment option for this disease. Therefore, it is obvious that the free and total PSA test used in diagnosis will be used to distinguish between PCa and BPH for a while. In terms of PCa, miR-21, miR-142, miR-143, miR-146a can contribute to the literature as well as guide developing PCa treatment strategies. But it has been shown that accurate and rapid diagnoses of PCa patients staying in the gray area cannot be possible with these miRNAs. These miRNAs should be investigated more widely to be used in screening for malignant-benign differentiation, to increase the diagnostic power of miRNAs, or to be used as a stronger marker alone or as a panel.

## Dodatak

### Acknowledgments

This work was supported by the Istanbul University-Cerrahpasa Research Fund (Project No: 22786)

### Data Availability Statement

The data that support the findings of this study are available from the corresponding author, I. Murat Bolayırlı upon request.

### Conflict of interest statement

All the authors declare that they have no conflict of interest in this work.
